# Patient-reported outcome measures for physical function in cancer patients: content comparison of the EORTC CAT Core, EORTC QLQ-C30, SF-36, FACT-G, and PROMIS measures using the International Classification of Functioning, Disability and Health

**DOI:** 10.1186/s12874-022-01826-z

**Published:** 2023-01-21

**Authors:** T Schurr, F Loth, E Lidington, C Piccinin, JI Arraras, M Groenvold, B Holzner, M van Leeuwen, MA Petersen, H Schmidt, T Young, JM Giesinger, European Organisation for Research, Treatment of Cancer Quality of Life Group (EORTC QLG), Juan I. Arraras, Mogens Groenvold, Marieke van Leeuwen

**Affiliations:** 1 Department of Psychiatry, Psychotherapy, Psychosomatics, and Medical Psychology, University Hospital of Psychiatry I, Innsbruck Medical University, Anichstraße 35, A-6020 Innsbruck, Austria; 2 Professorship for Psychological Diagnostics and Intervention Psychology, Faculty of Philosophy and Education, Catholic University of Eichstätt-Ingolstadt, Ostenstraße 25, 85072 Eichstätt, Germany; 3 Cancer Behavioural Science Unit, King’s College London, Guy’s Hospital, St Thomas Street, London, SE1 9RT UK; 4 Quality of Life Department, EORTC, Avenue E. Mounier, 83/11, 1200 Brussels, Belgium; 5 Medical Oncology Department, Hospital Universitario de Navarra, C/Irunlarrea 3, S31008 Pamplona, Spain; 6 Palliative Care Research Unit, Department of Geriatrics and Palliative Medicine GP, Bispebjerg & Frederiksberg Hospital, University of Copenhagen, Copenhagen, Denmark; 7 Department of Psychiatry, Psychotherapy, Psychosomatics, and Medical Psychology, University Hospital of Psychiatry II, Innsbruck Medical University, Anichstraße 35, A-6020 Innsbruck, Austria; 8 Division of Psychosocial Research & Epidemiology, The Netherlands Cancer Institute, Plesmanlaan 121, 1066 CX Amsterdam, The Netherlands; 9 University Clinic and Outpatient Clinic for Radiotherapy and Institute of Health and Nursing Science, Medical Faculty of Martin Luther University Halle-Wittenberg, Halle (Saale), Germany; 10 Lynda Jackson Macmillan Centre, Mount Vernon Cancer Centre, Rickmansworth Rd, GB- HA6 2RN Halle (Saale), UK

**Keywords:** Patient-reported outcome measures, Physical function, Cancer, Linking, International classification of functioning disability and health, ICF, PROM

## Abstract

**Background:**

Patient-reported physical function (PF) is a key endpoint in cancer clinical trials. Using complex statistical methods, common metrics have been developed to compare scores from different patient-reported outcome (PRO) measures, but such methods do not account for possible differences in questionnaire content. Therefore, the aim of our study was a content comparison of frequently used PRO measures for PF in cancer patients.

**Methods:**

Relying on the framework of the International Classification of Functioning, Disability and Health (ICF) we categorized the item content of the physical domains of the following measures: EORTC CAT Core, EORTC QLQ-C30, SF-36, PROMIS Cancer Item Bank for Physical Function, PROMIS Short Form for Physical Function 20a, and the FACT-G. Item content was linked to ICF categories by two independent reviewers.

**Results:**

The 118 items investigated were assigned to 3 components (‘d – Activities and Participation’, ‘b – Body Functions’, and ‘e – Environmental Factors’) and 11 first-level ICF categories. All PF items of the EORTC measures but one were assigned to the first-level ICF categories ‘d4 – Mobility’ and ‘d5 – Self-care’, all within the component ‘d – Activities and Participation’. The SF-36 additionally included item content related to ‘d9 – Community, social and civic life’ and the PROMIS Short Form for Physical Function 20a also included content related to ‘d6 – domestic life’. The PROMIS Cancer Item Bank (v1.1) covered, in addition, two first-level categories within the component ‘b – Body Functions’. The FACT-G Physical Well-being scale was found to be the most diverse scale with item content partly not covered by the ICF framework.

**Discussion:**

Our results provide information about conceptual differences between common PRO measures for the assessment of PF in cancer patients. Our results complement quantitative information on psychometric characteristics of these measures and provide a better understanding of the possibilities of establishing common metrics.

**Supplementary Information:**

The online version contains supplementary material available at 10.1186/s12874-022-01826-z.

## Background

Physical function (PF) is a domain of health-related quality of life (HRQOL) that refers to activities that are fundamental to maintaining functional independence and influence independent living [1, 2].

PF is also a key endpoint in cancer clinical trials that plays a crucial role in the assessment of treatment efficacy [3, 4]. The importance of PF is well reflected in guidance documents of the European Medicines Agency [5] and the US Food and Drug Administration [6], and the domain is frequently part of drug-related patient-reported outcome (PRO) labelling claims [3].

Moreover, patient-reported PF has been shown to be an independent prognostic factor for overall survival across different types of cancer and disease stages [7], frequently exceeding the Eastern Cooperative Oncology Group (ECOG) performance status in prognostic value [8]. In studies determining health utilities for calculating quality-adjusted life years, PF was associated with the largest utility decrements, i.e. the largest impact on a patient’s evaluation when weighting HRQOL against life expectancy [9, 10].

A variety of well-validated multi-dimensional PRO measures and measurement systems are available that target PF and help to determine whether patients are able to perform tasks ranging from light activities to strenuous exercise. The most frequently used PRO measures in clinical trials [11] are the European Organisation for Research and Treatment of Cancer (EORTC) measures, the Functional Assessment of Chronic Illness Therapy (FACIT) measures, and the SF-36; and in particular for measuring PF, the EORTC QLQ-C30 and the SF-36 are used most often [12]. With the introduction of the Patient-Reported Outcomes Measurement Information System [PROMIS, 13], another option for the collection of PROs has been added. PROMIS, and the newly developed EORTC CAT Core [14, 15], are based on the concept of item banks. These rely on Item Response Theory (IRT) measurement models that enable the generation of computer-adaptive assessments and static short-forms tailored to specific settings [16].

The use of different PRO measures limits the comparability of study findings, even if the various measures assert to measure the same construct and are named similarly. To overcome this limitation common metrics have been introduced for certain PRO measures [17–19] that allow score conversion from one measure to another based on a variety of statistical methods. While the statistical methods are key for this, meaningful conversions of scores and pooling of such data should also account for differences and similarities of the content of the different PRO measures, i.e. they should consider whether the same aspects of PF are measured.

The items from the above-mentioned PRO measures assessing PF frequently relate to the ability to perform instrumental activities of daily living (e.g. self-care), but also to mobility, movement and fine motor skills. However, differences in the operationalization of PF may compromise comparability of scores from the different measures, even if statistical models may be developed for score conversion. The International Classification of Functioning, Disability and Health [ICF, 20] provides a framework for categorizing information on patients’ health and has frequently been used for comparing the item content of PRO measures in various medical fields [21–24].

This study is part of the first step of an ongoing EORTC QLG project, which aims to investigate the potential for linking scores from commonly used PRO measures in cancer research, with a focus on the EORTC CAT Core domains [14]. Information on item content differences and congruence is key for a better understanding of situations where common metrics enable meaningful conversions of scores from different PRO measures. Therefore, in the first phase, we aim to qualitatively assess the content of the different measurement tools to investigate possible conceptual (dis)similarities. In the second phase, quantitative analyses of the actual linkage of scores from these measures using statistical methods will be conducted. In this article, we present the results from the qualitative analysis comparing the item content of the physical domains of the following frequently used PRO measures using the ICF framework:

- EORTC CAT Core Physical Functioning item bank
- EORTC QLQ-C30 Physical Functioning scale
- SF-36 Physical Function scale
- PROMIS Short Form v2.0 Physical Function 20a
- PROMIS Cancer Item Bank v1.1 – Physical Function
- FACT-G Physical Well-being scale

## Methods

### Comparator measures for physical function

#### EORTC CAT Core and EORTC QLQ-C30

The EORTC CAT Core [14, 15] has been developed to measure the same 14 functional health domains and symptoms as the EORTC QLQ-C30 v3.0 [25], the most widely used PRO measure in cancer research [11, 26, 27]. It comprises item banks for each of the QLQ-C30 domains (with the exception of Global QOL). The item bank for PF consists of 31 items including the five PF items from the QLQ-C30 [28]. The additional items have been developed to fit conceptually and psychometrically with the QLQ-C30 PF items [28, 29]. For item bank development, ICF categories were used as well as the definition of PF according to Stewart and Kamberg [30], which defines PF as 'the performance of or capability to perform a variety of physical activities' such as 'bathing, dressing, walking, bending, climbing stairs, and running'.

The EORTC CAT Core item banks and the EORTC QLQ-C30 v3.0 use a 4-point rating scale as response format with categories ranging from ‘Not at all’ to ‘Very much’, without referring to a specific recall period.

#### SF-36

The Short Form 36 (SF-36) is a 36-item HRQOL measure [31, 32] that comprises eight individual domains including PF. The PF scale is conceptualized as the ‘performance of or capacity to perform a variety of activities that are normal for an individual in good health’ [33], including self-care, mobility, and physical activities. The questionnaire items assess limitations of physical activities and functioning due to health conditions. The PF domain consists of 10 items with a 3-point rating scale response format (response categories: ‘Yes, limited a lot’, ‘Yes, limited a little’, and ‘No, not limited at all’) and no specific recall period.

#### PROMIS cancer item bank v1.1 – physical function

The Patient-Reported Outcomes Measurement Information System (PROMIS) Cancer Item Bank v1.1 for PF is an item bank based on the PROMIS Physical Function Item Bank v1.0. The cancer-specific item bank includes 45 items, of which 8 items are uniquely cancer specific [34]. In general, domain mapping (e.g. physical function, including the conceptual framework and structure) in PROMIS was developed through independent literature reviews, a consensus-building Delphi process and statistical analysis concerning dimensionality of assessed health status. This process also embedded the WHO physical, mental and social framework, as well as the ICF framework and a 2-factor model of physical and mental health [35].

In PROMIS, PF is defined as ‘the ability to carry out various activities that require physical capability, ranging from self-care (basic activities of daily living) to more vigorous activities that require increasing degrees of mobility, strength, and/or endurance’ [36].

The questions that do not have a specific recall period are answered on a 5-point Likert scale ranging from ‘without any difficulty’ to ‘unable to do’ or ‘not at all’ to ‘cannot do’.

#### PROMIS – short form v2.0 – physical function 20a

The PROMIS Physical Function 20a [13] is a generic short-form based on the PROMIS Physical Function Item Bank v2.0 [34]. Six items from the PROMIS Cancer Item Bank v1.1 are included in the Physical Function 20a short-form. No recall period is specified in the short-form. The questions are answered on a 5-point Likert scale ranging from ‘Not at all’ to ‘Cannot do’ or ‘Without any difficulty’ to ‘Unable to do’.

#### FACT-G

The Functional Assessment of Cancer Therapy Scale – General (FACT-G, [37]) is a well-validated and commonly used questionnaire for the assessment of HRQOL in cancer patients. It provides scores for four domain scales including a physical well-being scale and a total score calculated from all four scales. Item generation was realised by interviewing patients and oncology specialists in a semi structured manner. The 27 items of the FACT-G version 4.0 [38] are rated on a 5-point Likert scale ranging from ‘Not at all’ to ‘Very much’, referring to a seven day recall period. The physical well-being scale consists of seven items that assess e.g. common symptoms in cancer patients such as fatigue or pain, a question on side-effects of treatment, and a question on being bedbound.

#### Linking of item content to the ICF framework

The ICF introduced in 2001 [20] by the World Health Organization (WHO) provides a unified standard language and conceptual framework for the description of health and health-related well-being. It has a hierarchical structure that consists of four components coded with letters (‘b – Body function’, ‘s – Structure’, ‘d – Activities and Participation’, and ‘e – Environmental factors”) and further levels of (sub)categories coded with numbers.

According to the ICF classification, the letters representing the components are followed by numbers, with the first digit indicating the first-level (chapter) category, the second and third digit indicating the second-level category, and the fourth digit stating the third-level category. Examples for the hierarchical structure of the ICF categories are given in Table 1.

**Table 1:** Examples of the hierarchical structure of the International Classification of Functioning, Disability and Health (ICF) categories (WHO, 2001)

Code	Description	Level
d	Activities and participation	Component
d5	Self-care	First level (Chapter)
d540	Dressing	Second level
d5400	Putting on clothes	Third level
b	Body functions	Component
b4	Functions of the cardiovascular, haematological, immunological and respiratory systems	First level (Chapter)
b455	Exercise tolerance functions	Second level
b4550	General physical endurance	Third level

To compare the item content of the PF measures we relied on the ICF version 2001 [20] and the methodology introduced by Cieza et al. [39–41]. The authors have established linking rules that define how to link questionnaire items covering different ICF categories, content not included in the ICF, or content not explicitly named in a corresponding ICF category. These linking rules have been used widely in the literature to compare the content of different PRO measures assessing a wide range of domains [21–24].

In an initial step of linking item content to the ICF classification, meaningful concepts are identified in each item of the questionnaires. Next, these concepts are mapped onto corresponding third-level ICF categories, i.e. assigned to ICF codes. If an item contains more than one meaningful concept, these are linked separately to the respective ICF categories, i.e. a single item may be linked to multiple ICF categories (e.g. PROMIS PF Short-form 20a Item No 3: ‘Are you able to dress yourself, including tying shoelaces and buttoning your clothes?’ was linked to d440 ‘Fine hand use’, and d540 ‘Dressing’). According to the recommendations by Cieza et al. [39–41], meaningful concepts of items that cannot be represented by ICF categories are coded as not covered (nc) if the concept is outside the ICF framework, and as not defined (nd) if the concept cannot be assigned to a component.

As part of the linking procedure each individual item in the PRO measures under investigation was coded independently by two of the following reviewers: FL, EL, CP, and JMG. Disagreements between two reviewers were discussed to reach consensus and another reviewer of the above was consulted to help resolving disagreements if needed.

Following the linking of item content, we analysed descriptively for each PRO measure under investigation, the number of codings per ICF category, the number of items (not) covered by ICF categories, and the number of different ICF categories covered by each PRO measures.

## Results

Across all PRO measures under investigation the 118 PF items were assigned to 3 components (‘d – Activities and Participation’, ‘b – Body Functions’, and ‘e – Environmental Factors’), 11 first-level ICF categories, 30 s-level categories, and 69 third-level categories. Four concepts were categorized as ‘not covered’ or ‘not definable’ within the ICF framework.

The 31 items of the EORTC CAT Core PF item bank were assigned to 14 different second-level categories in ‘d – Activities and Participation’ and to 1 s-level category in ‘e – Environmental Factors’ (an item referring to a walking aid). Within ‘d – Activities and Participation’, all items belonged to the first-level categories ‘d4 – Mobility’ and ‘d5 – Self-care’. The most frequently found second-level categories were ‘d430 – Lifting and carrying objects’ (10 codings), ‘d450 – Walking’ (8 codings), ‘d455 – Moving around’ (7 codings), and ‘d520 – Caring for body parts’ (6 codings).

The five items of the EORTC QLQ-C30 PF scale (which are part of the EORTC CAT Core PF item bank) covered 8 different second-level categories, all within the component ‘d – Activities and Participation’ (6 codings in ‘d4 – Mobility’ and 4 codings in ‘d5 – Self-care’). The most common second-level categories were ‘d415 – Maintaining a body position’ and ‘d450 – Walking’ (2 codings each).

The SF-36 covered 9 different second-level categories with its 10-item PF scale, all of them being part of the component ‘d – Activities and Participation’. Fifteen codings were in ‘d4 – Mobility’, 2 codings in ‘d5 – Self-care’, and 2 codings in ‘d9 – Recreation and leisure’. The second-level categories ‘d430 – Lifting and carrying objects’ (4 codings), ‘d450 – Walking’, and ‘d455 – Moving around’ (3 codings each) were assigned most often.

The cancer-specific PROMIS item bank for PF covered 19 different second-level categories in the component ‘d – Activities and Participation’ and 1 s-level category (‘b455 – Exercise tolerance functions’; 3 codings) in the component ‘b – Body functions’. Within the component ‘d – Activities and participation’, the item bank covered the first-level categories ‘d2 – General tasks and demands’ (1 coding), ‘d4 – Mobility’ (45 codings), ‘d5 – Self-care’ (9 codings), ‘d6 – Domestic life’ (14 codings), and ‘d9 – Community, social and civic life’ (7 codings). The most frequently found second-level categories were ‘d430 – Lifting and carrying objects’ (15 codings), ‘d640 – Doing housework’ (11 codings), and ‘d455 – Moving around’ (8 codings).

The PROMIS PF Short Form 20a covered 15 different second-level categories, all of them being part of the component ‘d – Activities and Participation’. These second-level categories belonged to ‘d4 – Mobility’ (20 codings), ‘d5 – Self-care’ (6 codings), ‘d6 – Domestic life’ (3 codings), and ‘d9 – Community, social and civic life’ (1 coding). The most frequently found individual second-level categories were ‘d410 – Changing basic body position’ (5 codings), ‘d510 – Washing oneself’ (5 codings), and ‘d430 – Lifting and carrying objects’ (4 codings).

The FACT-G Physical Wellbeing scale with its 7 items covered six different second-level categories in the components ‘b – Body function’ (4 codings) and ‘d – Activities and participation’ (2 codings) and contained four concepts that could not be assigned to ICF categories. The six second-level categories were: ‘b130 – Energy and drive functions’, ‘b289 – sensation of pain, other and unspecified’, ‘b455 – Exercise tolerance functions’, ‘b535 – Sensations associated with the digestive system’, ‘d415 – Maintaining a body position’, and ‘d760 – Family relationships’.

The percentage of codings in the component ‘d – Activities and participation’ was largest for the EORTC QLQ-C30 (10 out of 10 codings; 100%), the PROMIS Short Form 20a (30/30; 100%) and the SF-36 (19/19; 100%) followed by the EORTC CAT Core (48/49; 98.0%), the cancer-specific PROMIS item bank for PF (76/79; 96.2%), and the FACT-G (2/10; 20%). Within the component ‘d – Activities and participation’ the first-level category ‘d4 – Mobility” was representing more than half of the coded item content for all PRO measures (but the FACT-G): SF-36 (15/19; 78.9%), PROMIS Short Form 20a (20/30; 66.7%), EORTC CAT Core (32/49 codings; 65.3%), EORTC QLQ-C30 (6/10; 60.0%), cancer-specific PROMIS item bank for PF (45/79%; 57.0%), and FACT-G (1/10; 10%). The category ‘d5 – Self-care’ was the second most coded first-level category for the EORTC QLQ-C30 (4/10; 40.0%) and the EORTC CAT Core (16/49; 32.7%), while it was less commonly found for the PROMIS Short Form 20a (6/30; 20.0%), the cancer-specific PROMIS item bank for PF (9/79%; 11.4%), the SF-36 (2/19; 10.5%), and the FACT-G (1/10; 10%). The first-level category ‘d6 – Domestic life” was coded only for the PROMIS measures: cancer-specific PROMIS item bank for PF (14/79; 17.7%) and PROMIS Short Form 20a (3/30; 10%), while the first-level category ‘d9 – Community, social and civic life’ was also relevant to the SF-36: SF-36 (2/19; 10.5%), PROMIS Short Form 20a (1/30; 3.3%), and cancer-specific PROMIS item bank for PF (7/79; 8.9%).

**Fig. 1:**
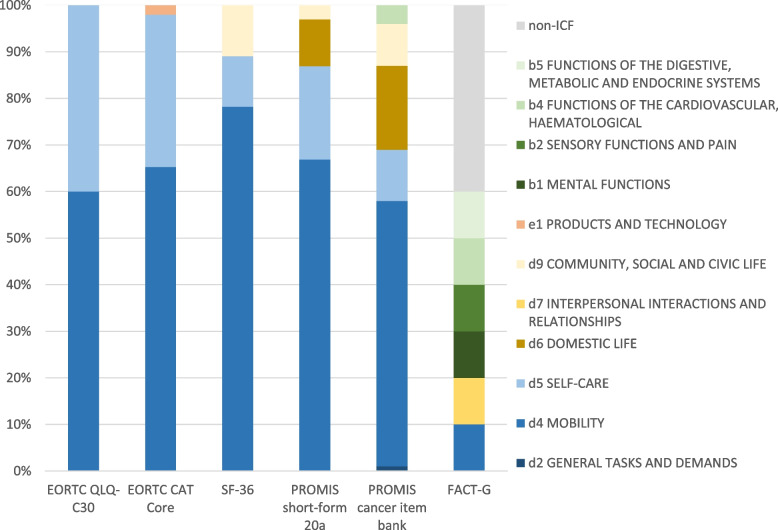
Bar graph showing the proportions of International Classification of Functioning, Disability and Health (ICF) categories that have been linked within the respective patient-reported outcome measures for physical function

Further details on the PF domain of all PRO measures under investigation are shown in Table 2 and Table 3 and Fig. 1. Results for third-level categories are reported in Supplementary Table S1. When interpreting the descriptive statistics in the tables and figure, please note that multiple third-level ICF categories could be assigned to each item of the respective measure. For example the item 4 ‘Do you have any trouble walking up a flight of stairs?’ from the EORTC CAT Core PF was assigned to d450 ‘Walking’, and d455 ‘Moving around’.

**Table 2:** Comparison of the characteristics of the PRO measures under investigation

	EORTC CAT Core	EORTC QLQ-C30	SF-36	PROMIS	PROMIS	FACT-G
	PF item bank	PF scale	PF scale	PF short-form 20a	PF cancer item bank	Physical Well-being scale
Number of items in questionnaire	31	5	10	20	45	7
Number of items that are covered by ICF categories	31	5	10	20	45	4
Number of different first-level ICF categories	3	2	3	4	6	6
Number of different second-level ICF categories	14	8	9	15	20	6
Number of different third-level ICF categories	30	10	13	23	39	6
Total number of third-level ICF categories assigned to items	49	10	19	30	79	6

**Table 3:** Number of third-level categories reported within each first- and second-level category of the International Classification of Functioning, Disability and Health (ICF) represented in the PRO measures under investigation

ICF Categories	EORTC CAT Core	EORTC QLQ-C30	SF-36	PROMIS	PROMIS	FACT-G
**Component** *First level* *Second level*	PF item bank	PF scale	PF scale	PF short-form 20a	PF cancer item bank	Physical Well-being scale
b BODY FUNCTIONS					3	4
b1 MENTAL FUNCTIONS						1
b130 Energy and drive functions						1
b2 SENSORY FUNCTIONS AND PAIN						1
b289 Sensation of pain, other and unspecified						1
b4 FUNCTIONS OF THE CARDIOVASCULAR, HAEMATOLOGICAL, IMMUNOLOGICAL AND RESPIRATORY SYSTEMS					3	1
b455 Exercise tolerance functions					3	1
b5 FUNCTIONS OF THE DIGESTIVE, METABOLIC AND ENDOCRINE SYSTEMS						1
b535 Sensations associated with the digestive system						1
d ACTIVITIES AND PARTICIPATION	48	10	19	30	76	2
d2 GENERAL TASKS AND DEMANDS					1	
d230 Carrying out daily routine					1	
d4 MOBILITY	32	6	15	20	45	1
d410 Changing basic body position	1		2	5	7	
d415 Maintaining a body position	2	2		1	3	1
d420 Transferring oneself				1		
d430 Lifting and carrying objects	10	1	4	4	15	
d440 Fine hand use	1			2	1	
d445 Hand and arm use			1	1	7	
d450 Walking	8	2	3	1	2	
d455 Moving around	7		3	3	8	
d460 Moving around in different locations	3	1				
d498 Mobility, other specified			2	2	2	
d5 SELF-CARE	16	4	2	6	9	
d510 Washing oneself	2	1	1	5	2	
d520 Caring for body parts	6				1	
d530 Toileting	1	1			1	
d540 Dressing	4	1	1	1	4	
d550 Eating	2	1			1	
d599 Self-care unspecified	1					
d6 DOMESTIC LIFE				3	14	
d620 Acquisition of goods and services					1	
d640 Doing housework				1	11	
d649 Household tasks, other specified and unspecified				1		
d650 Caring for household objects				1	1	
d698 Domestic life, other specified					1	
d7 INTERPERSONAL INTERACTIONS AND RELATIONSHIPS						1
d760 Family relationships						1
d9 COMMUNITY, SOCIAL AND CIVIC LIFE			2	1	7	
d920 Recreation and leisure			2	1	7	
e ENVIRONMENTAL FACTORS	1					
e1 PRODUCTS AND TECHNOLOGY	1					
e120 Products and technology for personal indoor and outdoor mobility and transportation	1					
nc (not covered)						1
nd-func (functioning)						1
nd-ph (physical health)						2

Third-level ICF categories were assigned to each meaningful concept in an item, thus, the total of classifications could exceed the total number of items

## Discussion

In our analysis, we classified and compared the item content of six commonly used PRO measures for PF in cancer patients relying on the ICF reference system established by the WHO to assess concept equivalence, i.e. the degree to which they measure the same concepts.

The vast majority of the items (97%) could be assigned to ICF categories within the component ‘d—Activities and Participation’. For all PRO measures but the FACT-G, the first-level category ‘d4 – Mobility’ covered more than half of the item content, followed by ‘d5 – Self-care’ for the EORTC measures, the SF-36, and the PROMIS Short Form 20a. For the cancer-specific PROMIS item bank ‘d6 – Domestic life’ was the second most frequently used first-level category. The FACT-G Physical Well-being was the most heterogeneous measure with items related to ‘d – Activities and Participation’, ‘b – Body Functions’, and content that could not be categorized within the ICF framework.

The most relevant difference between the EORTC and the PROMIS measures was found for the first-level categories ‘d6—domestic life’ and ‘d9—community, social and civic life’ that were covered by the PROMIS, but not by the EORTC measures. This finding reflects differences in the overall EORTC and PROMIS measurement systems. The EORTC QLQ-C30 has distinct scales for physical, role, and social functioning, and this distinction has been maintained by the EORTC CAT Core, that provides individual item banks to measure role functioning [14, 15, 42] and social functioning [14, 15]. Therefore first-level categories such as d6 (Domestic life) or d9 (Community, social and civic life) have not been included in the EORTC physical functioning item bank. In quantitative analyses, the EORTC role functioning domain has been shown to be a domain that is strongly associated with, but still distinguishable from, the PF domain [43]. However, in the literature is still a debate on conceptual differences between physical and role functioning [44]. In the PROMIS framework a distinct role function measure is not available, since the concepts of role functioning and social functioning are combined in the item bank v2.0 Ability to Participate in Social Roles and Activities (e.g. measuring social function and social relationships; [45]).

The finding that the EORTC CAT Core includes more items related to the self-care ICF domain, i.e., items assessing low levels of physical function, than the comparator measures possibly reflects the focus on cancer populations and the development of the item bank in cooperation with the European Palliative Care Research Collaborative [29].

For the PROMIS measures, a content analysis using the ICF has been conducted previously by Tucker et al. [46] who, in line with our analysis, reported that most of the item content was related to d4 ‘Mobility’ and d5 ‘Self-care’ in the ‘Activities and Participation’ component and coverage of other first-level categories such as d6 ‘Domestic Life’ or d9 ‘Community, Social and Civic Life’ was limited. Consistent with our results a low number of items was also found to be related to “Body Functions”. Furthermore, our results are also in accordance with the analysis of the SF-36 by the original authors of the linking methodology [39] who also found the “Activities and Participation” component to cover all the content of the PF scale.

The physical well-being domain of the FACT-G assesses, for example, pain, reduced energy, and feelings of illness or impairments caused by side effects of the therapy, thus providing an assessment of general symptom burden rather than assessing PF specifically. Differences between the EORTC QLQ-C30 and the FACT-G domains have been highlighted previously [47, 48], and while the scale name itself reflects the conceptual difference, we decided to include this scale in our analysis since it has been linked to PF measures previously [49].

For the cancer-specific PROMIS PF item bank and the EORTC CAT Core, the results from our content analysis may also be used for content balancing in computer-adaptive assessments [50] or when creating short-forms or sub-sets from item banks [51] for physical function. Content balancing methods allow the selection of items not only based on psychometric criteria (e.g. maximum information) but also ensure that all important aspects of PF are adequately covered in an assessment. Content balancing may help to improve measurement accuracy and support consistent item selection [52].

A major challenge for a conceptual comparison of measures for PF is the lack of a comprehensive conceptual framework that explicitly defines PF. Despite previous efforts [2, 53] no single widely accepted and applicable definition has yet been established (which may at least in part result from the different purposes and perspectives when measuring PF). For our analysis, we used the ICF as this is a common framework for content comparisons of PRO measures and provides very detailed categories for the analysis of PF. However, we would like to emphasise that the type of framework used necessarily impacts on the assessment of similarities and differences between PRO measures. A further limitation of this study is the selection of PRO measures for PF that we analyzed. We did not rely on a systematic review for identifying all such measures applied in cancer patients but selected only those most frequently used in clinical research [11, 26, 27] and clinical practice [54] with the additional inclusion of PROMIS measures that are quickly gaining importance for PRO measurement across medical fields [55, 56]. For PROMIS we only investigated two measures of physical function, and content from other PROMIS short-forms may differ and include/exclude specific ICF categories. Furthermore, our analysis only focused on the comparison of item content but did not consider psychometric characteristics and the measurement range, i.e. the coverage of low, medium, and high levels of PF by the various measurement instruments. While such information is available from previous studies [13–15, 57], the levels of PF were not investigated in this analysis, which was based on the ICF framework.

In a next step in our ongoing project, we will evaluate scale equivalence with quantitative methods, such as regression models, IRT models, or equipercentile equating [58], to assess the possibilities of equating PF scores from the EORTC CAT Core and other PRO measures included in this analysis. The comparison of PRO measures based on the ICF classifications in this study will add to the results from applying quantitative methods to decide for which PRO measures linking scores or creating a common metric is meaningful.

In conclusion, the results from our analysis may inform clinicians and researchers when selecting the optimal PRO measure for PF in a specific study or patient population. With regard to linking of PRO measures we expect that the shared content related to mobility and self-care, which was predominant in the physical domain of all measures but the FACT-G, suggests that conceptually meaningful linking can be conducted, and quantitative methods are likely to allow for sufficient precision of such procedures.

## Supplementary Information

**Additional file1**. **Table S1**. Number of third-level categories of ICF represented in the PRO measures under investigation

## Data Availability

The datasets used and/or analysed during the current study available from the corresponding author on reasonable request.
