# Supplementary material for: Patient-reported outcome measures for physical function in cancer patients: content comparison of the EORTC CAT Core, EORTC QLQ-C30, SF-36, FACT-G, and PROMIS measures using the International Classification of Functioning, Disability and Health
Source: BMC Med Res Methodol. 2023 Jan 21;23:21. doi: 10.1186/s12874-022-01826-z (PMC9862545; doi:10.1186/s12874-022-01826-z)
Supplement: Supplementary file 1 — Additional file1. Table S1. Number of third-level categories of ICF represented in the PRO measures under investigation [file 12874_2022_1826_MOESM1_ESM.docx]

Supplementary Table S1: Number of third-level categories of ICF represented in the PRO measures under investigation

| ICF Categories | EORTC CAT Core | EORTC QLQ-C30 | SF-36 | PROMIS | PROMIS | FACT-G |
| --- | --- | --- | --- | --- | --- | --- |
| Third level | PF item bank | PF scale | PF scale | PF short-form 20a | Cancer item bank | Physical Well-being scale |
| b1300 Energy level |  |  |  |  |  | 1 |
| b289 sensation of pain, other and unspecified |  |  |  |  |  | 1 |
| b4550 General physical endurance |  |  |  |  | 3 |  |
| b4552 Fatiguability |  |  |  |  |  | 1 |
| b5350 Sensation of nausea |  |  |  |  |  | 1 |
| d2301 Managing daily routine |  |  |  |  | 1 |  |
| d4100 Lying down |  |  |  |  | 1 |  |
| d4101 Squatting |  |  |  |  | 1 |  |
| d4102 Kneeling |  |  | 1 | 1 | 1 |  |
| d4103 Sitting |  |  |  | 1 |  |  |
| d4104 Standing |  |  |  | 1 |  |  |
| d4105 Bending | 1 |  | 1 | 1 | 2 |  |
| d4108 Changing basic body position, other specified |  |  |  | 1 | 2 |  |
| d4150 Maintaining a lying position | 1 | 1 |  |  |  | 1 |
| d4153 Maintaining a sitting position | 1 | 1 |  | 1 |  |  |
| d4154 Maintaining a standing position |  |  |  |  | 3 |  |
| d4200 Transferring oneself while sitting |  |  |  | 1 |  |  |
| d4300 Lifting | 3 |  | 3 | 2 | 6 |  |
| d4301 Carrying in the hands | 1 |  | 1 | 2 | 9 |  |
| d4303 Carrying on shoulders, hip and back | 1 |  |  |  |  |  |
| d4305 Putting down | 1 |  |  |  |  |  |
| d4309 Lifting and carrying objects, unspecified | 4 | 1 |  |  |  |  |
| d4400 Picking up | 1 |  |  |  | 1 |  |
| d4402 Manipulating |  |  |  | 2 |  |  |
| d4450 Pulling |  |  |  |  | 1 |  |
| d4451 Pushing |  |  | 1 | 1 | 5 |  |
| d4458 Hand and arm use, other specified |  |  |  |  | 1 |  |
| d4500 Walking short distances | 3 | 1 | 2 |  | 1 |  |
| d4501 Walking long distances | 3 | 1 | 1 | 1 |  |  |
| d4502 Walking on different surfaces | 2 |  |  |  | 1 |  |
| d4551 Climbing | 3 |  | 2 | 1 | 4 |  |
| d4552 Reaching | 1 |  |  |  |  |  |
| d4552 Running | 3 |  | 1 | 2 | 4 |  |
| d4602 Moving around outside the home and other buildings | 3 | 1 |  |  |  |  |
| d498 Mobility, other specified |  |  | 2 | 2 | 2 |  |
| d5100 Washing body parts | 1 |  |  | 2 |  |  |
| d5102 Drying oneself |  |  |  | 2 |  |  |
| d5109 Washing oneself, unspecified | 1 | 1 | 1 | 1 | 2 |  |
| d5201 Caring for teeth | 1 |  |  |  |  |  |
| d5202 Caring for hair | 1 |  |  |  | 1 |  |
| d5203 Caring for fingernails | 1 |  |  |  |  |  |
| d5204 Caring for toenails | 2 |  |  |  |  |  |
| d5208 Caring for body parts, other specified [fee | 1 |  |  |  |  |  |
| d5309 Toileting, unspecified | 1 | 1 |  |  | 1 |  |
| d5400 Putting on clothes | 2 |  | 1 | 1 |  |  |
| d5401 Taking off clothes | 1 |  |  |  |  |  |
| d5402 Putting on footwear |  |  |  |  | 1 |  |
| d5403 Taking off footwear |  |  |  |  | 1 |  |
| d5409 Dressing, unspecified | 1 | 1 |  |  | 2 |  |
| d550 Eating | 2 | 1 |  |  | 1 |  |
| d599 Selfcare unspecified | 1 |  |  |  |  |  |
| d6200 Shopping |  |  |  |  | 1 |  |
| d6401 Cleaning cooking area and utensils |  |  |  |  | 1 |  |
| d6402 Cleaning living area |  |  |  |  | 3 |  |
| d6403 Using household appliances |  |  |  | 1 | 3 |  |
| d6405 Disposing of garbage |  |  |  |  | 1 |  |
| d6408 Doing housework, other specified |  |  |  |  | 3 |  |
| d649 Household tasks, other specified and unspecified |  |  |  | 1 |  |  |
| d6505 Taking care of plants, indoors and outdoors |  |  |  | 1 | 1 |  |
| d698 Domestic life, other specified |  |  |  |  | 1 |  |
| d7608 Family relationships, other specified |  |  |  |  |  | 1 |
| d9200 Play |  |  |  |  | 1 |  |
| d9201 Sports |  |  | 2 | 1 | 2 |  |
| d9203 Crafts |  |  |  |  | 1 |  |
| d9204 Hobbies |  |  |  |  | 2 |  |
| d9208 Recreation and leisure, other specified |  |  |  |  | 1 |  |
| e1201 Assistive products and technology for personal indoor and outdoor mobility and transportation | 1 |  |  |  |  |  |
| nc (not covered) |  |  |  |  |  | 1 |
| nd-func (functioning) |  |  |  |  |  | 1 |
| nd-ph (physical health) |  |  |  |  |  | 2 |

ICF = International Classification of Functioning, Disability and Health
